# Sex Differences in Cardiovascular Outcomes of Intravascular Imaging-Guided PCI: A Meta-Analysis of Randomized Controlled Trials

**DOI:** 10.1016/j.jacadv.2025.102076

**Published:** 2025-08-18

**Authors:** Chidubem Ezenna, Sammudeen Ibrahim, Prasana Ramesh, Mahmoud Ismayl, Mrinal Murali Krishna, Meghna Joseph, Kuan-Yu Chi, S. Elissa Altin, Michael G. Nanna, Andrew M. Goldsweig

**Affiliations:** a Department of Medicine, University of Massachusetts – Baystate Medical Center, Springfield, Massachusetts, USA; b Department of Cardiovascular Medicine, Mayo Clinic, Phoenix, Arizona, USA; c Department of Cardiovascular Medicine, Mayo Clinic, Rochester, Minnesota, USA; d Department of Medicine, Medical College Thiruvananthapuram, Trivandrum, Kerala, India; e Department of Medicine, Jacobi Medical Center, Albert Einstein College of Medicine, Bronx, New York, USA; f Section of Cardiovascular Medicine, Yale School of Medicine, New Haven, Connecticut, USA; g Department of Cardiovascular Medicine, Baystate Medical Center and Division of Cardiovascular Medicine, University of Massachusetts-Baystate, Springfield, Massachusetts, USA

**Keywords:** sex differences, cardiovascular outcomes, intravascular imaging, percutaneous coronary intervention, intravascular ultrasound, optical coherence tomography

## Abstract

**Background:**

Disparities in cardiovascular disease presentation and outcomes between men and women are well-documented. While intravascular imaging (IVI) improves percutaneous coronary intervention (PCI) outcomes, its potential sex-specific benefits remain unclear.

**Objectives:**

The purpose of this study was to determine sex differences in adverse cardiovascular events in coronary artery disease patients undergoing PCI with IVI guidance vs angiography alone.

**Methods:**

Systematic review of PubMed, Scopus, and Cochrane databases was conducted to identify randomized controlled trials comparing major adverse cardiovascular events (MACE) in men and women with coronary artery disease (presenting with acute or chronic coronary syndrome) undergoing IVI-guided vs angiography-guided PCI. Risk ratios (RRs) with 95% CIs were calculated using random-effects models.

**Results:**

Eight randomized controlled trials, comprising 14,812 patients (76.1% men and 23.9% women) were included. IVI-guided PCI significantly reduced MACE in both men (RR: 0.69; 95% CI: 0.58-0.81; *P* < 0.001) and women (RR: 0.64; 95% CI: 0.49-0.82; *P* < 0.001) compared with angiography alone (*P*_interaction_ = 0.62). Intravascular ultrasound–guided PCI reduced MACE in men and women compared with angiography alone (*P*_interaction_ = 0.86). Optical coherence tomography–guided PCI reduced MACE in men but not in women compared with angiography alone (*P*_interaction_ = 0.86).

**Conclusions:**

Despite the underrepresentation of women in IVI-guided PCI trials, this meta-analysis demonstrates that IVI-guided PCI confers a comparable reduction in MACE for both men and women, suggesting its potential to mitigate the long-standing sex-specific disparities in coronary intervention outcomes and supports its broader implementation in clinical practice.

Cardiovascular disease remains a leading cause of morbidity and mortality globally, with coronary artery disease (CAD) being a major contributor.^1^ For patients with CAD, percutaneous coronary intervention (PCI) is a cornerstone treatment modality.^1^ Technological advancements have led to the development of 2 major intravascular imaging (IVI) modalities, intravascular ultrasound (IVUS) and optical coherence tomography (OCT), which enhance PCI by offering high-resolution IVI of coronary arteries. These IVI tools enable a more precise assessment of lesion morphology, accurate stent sizing, and optimal stent deployment, ultimately improving procedural outcomes.^2^ However, despite the demonstrated clinical benefits of IVI in prior meta-analyses,^3^^,^^4^ it remains unclear whether these advantages extend equally to both sexes.

Women with CAD face unique modifiable and nonmodifiable challenges that can adversely impact their overall cardiovascular outcomes. Multiple studies have shown that women are more likely than men to be underdiagnosed, experience treatment delays, and have poorer outcomes after coronary revascularization.^5^, ^6^, ^7^ Innate biological differences, such as smaller coronary artery size, plaque morphology, and distinct pathophysiological mechanisms common in women, may partly explain these disparities.^8^ Moreover, the differences in clinical presentation and potential disparities in health care delivery may further contribute to the challenges women face in accessing equitable care.^7^ These factors emphasize the need for a more nuanced understanding of sex-specific differences in procedural outcomes and whether women benefit equally from IVI-guided PCI.

While current U.S. guidelines recommend IVI to guide PCI for all acute coronary syndrome (ACS) patients with complex lesions irrespective of sex,^9^ most randomized controlled trials (RCTs) on IVI have focused on the general population, with limited focus on sex-specific outcomes.^10^, ^11^, ^12^ Although prior studies have reported similar use of IVI between men and women after adjusting for potential confounders,^13^ women remain underrepresented in IVI-guided PCI trials.^10^, ^11^, ^12^ This underrepresentation may raise concerns about the generalizability of findings and whether women derive the same degree of benefit from IVI-guided PCI as men. To address this gap, we conducted a systematic review and meta-analysis of RCTs comparing IVI-guided vs angiography-guided PCI alone in treating CAD patients with either ACS or chronic coronary syndrome. We aimed to assess whether the cardiovascular benefits of IVI-guided PCI were consistent across both sexes, particularly assessing whether women derived similar reductions in adverse cardiovascular outcomes compared to men.

## Methods

### Protocol and registration

This systematic review and meta-analysis adhered to the PRISMA (Preferred Reporting Items for Systematic Reviews and Meta-Analyses) 2020 guidelines^14^ and was registered with the International Prospective Register of Systematic Reviews (PROSPERO) under registration number CRD42025642210. The study was deemed exempt by the University of Massachusetts-Baystate Medical Center Institutional Review Board, as all data used for analysis were publicly available and deidentified.

### Data sources and search strategy

Two authors (C.E. and S.I.) independently and comprehensively queried PubMed, Scopus, and Cochrane Central Registry of Controlled Trials databases from their inception through January 2025 to identify RCTs comparing the efficacy of IVI- (IVUS or OCT) vs angiography-guided PCI for ACS or chronic coronary syndrome. Both Medical Subject Headings and free-text terms were used to ensure broad coverage. Key search terms included “optical coherence tomography,” “intravascular ultrasound,” “intravascular imaging,” “angiography,” “percutaneous coronary intervention,” “stent,” and “randomized controlled trials.” No restrictions were placed on language, publication date, or sample size. The full search strategies for each database are provided in Supplemental Table 1.

### Endpoints

The primary endpoint was major adverse cardiovascular events (MACE), as defined by individual studies. Analyses were conducted to compare sex-specific differences and interaction in MACE among patients undergoing IVI-guided vs angiography-guided PCI. Subgroup analyses were conducted to compare sex-specific differences and interaction in MACE among patients undergoing IVUS-guided and OCT-guided PCI vs angiography alone. A separate subgroup analysis for patients who underwent PCI of complex lesions was also performed.

### Defining complex lesions

Complex coronary lesions were defined by the individual studies as those classified as type B2 or type C per the modified American College of Cardiology/American Heart Association classification system,^15^ as well as lesions located in anatomically challenging segments identified by the SYNTAX (Synergy Between PCI with Taxus and Cardiac Surgery) score.^16^ These included lesions with features such as bifurcation or trifurcation involvement, chronic total occlusions, ostial location, severe calcification, lesion length >20 mm, marked tortuosity or angulation (>45°), diffuse vessel disease, left main coronary artery involvement, and diffuse or multifocal in-stent restenosis.

### Study selection and eligibility criteria

Two authors (C.E. and S.I.) independently screened and reviewed the identified studies. Discrepancies were resolved through discussion or consultation with a third author (P.R.). Studies were eligible for inclusion in the primary analysis if they met the following criteria: 1) RCTs comparing IVUS- or OCT-guided PCI to angiography-guided PCI; 2) use of drug-eluting stents (DES); and 3) reporting sex-specific subgroup data for MACE. Exclusion criteria included: 1) nonrandomized studies, observational studies, conference abstracts, or case reports; and 2) studies that did not report sex-specific subgroup data for MACE. Studies utilizing IVUS or OCT were included regardless of lesion complexity or the clinical indication for PCI (ACS or chronic coronary syndrome). However, studies that failed to report sex-specific subgroup data were excluded. Additionally, studies that randomized patients to receive IVI alongside alternative imaging or physiology-based guidance methods other than angiography were excluded. To evaluate sex-based enrollment trends over time, all RCTs comparing IVI to angiography guided-PCI with DES were included in a separate trend analysis regardless of whether sex-specific MACE outcomes were reported. Attempts were made to contact authors for sex subgroup data, where data were not readily available.

### Data extraction and evaluation of study quality

Two authors (C.E. and S.I.) independently extracted data using prespecified tables. Extracted variables included baseline characteristics, definitions of MACE, event counts, and IVI modality used (IVUS or OCT). The methodological quality of the included studies was assessed using the Cochrane Collaboration’s Risk of Bias Tool for RCTs.^17^

### Data synthesis and statistical analysis

All statistical analyses were performed using Cochrane Review Manager (RevMan) software, version 5.3 (The Nordic Cochrane Centre, The Cochrane Collaboration, 2014). Mantel-Haenszel risk ratios (RRs) with 95% CIs were calculated to assess the outcomes of interest. Statistical heterogeneity was evaluated using Higgins’ I^2^ statistic, with values >50% considered indicative of moderate or higher heterogeneity.^18^ A random-effects model was used to calculate pooled effect estimates, employing the DerSimonian-Laird method to account for study-level variance.^19^ Publication bias was assessed using visual inspection of funnel plots. Trend analysis was performed using Microsoft Excel version 2505. For studies published in the same year, a weighted mean, based on the number of participants in each study, was used to generate a single annual estimate.

## Results

### Systematic review

**Figure 1:**
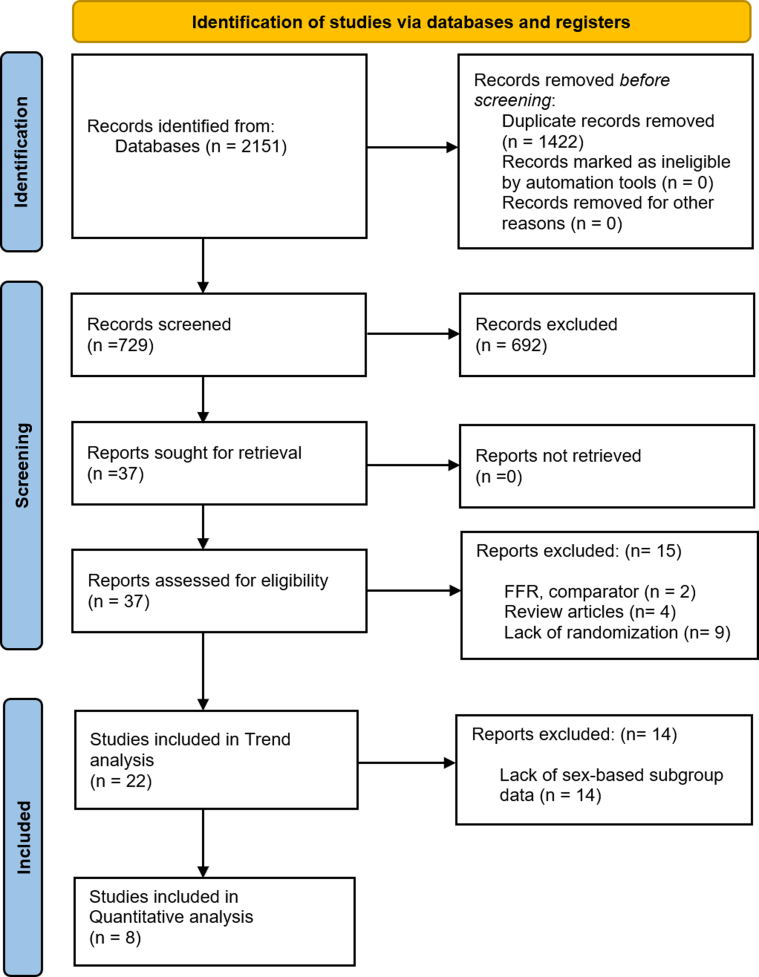
PRISMA Flow Diagram Showing the Selection Process of Included Studies PRISMA = Preferred Reporting Items for Systematic Reviews and Meta-Analyses.

The initial database search identified 2,151 studies. After removing 1,422 duplicates and screening titles and abstracts, 692 studies were excluded for not meeting our inclusion criteria. The remaining 37 studies underwent a full-text review, which resulted in the exclusion of 15 additional studies. The FLAVOUR (Fractional Flow Reserve And IVUS for Clinical Outcomes in Patients With Intermediate Stenosis; NCT02673424) and FORZA (FFR or OCT Guidance to Revascularize Intermediate Coronary Stenosis Using Angioplasty; NCT01824030) trials studied IVUS and OCT, respectively, and were excluded for utilizing FFR guidance in the control arm.^20^^,^^21^ Ultimately, 8 studies were included in the quantitative analysis,^22^, ^23^, ^24^, ^25^, ^26^, ^27^, ^28^, ^29^ while 22 studies contributed to the trend analysis.^12^, ^13^, ^14^^,^^22^, ^23^, ^24^, ^25^, ^26^, ^27^, ^28^, ^29^, ^30^, ^31^, ^32^, ^33^, ^34^, ^35^, ^36^, ^37^, ^38^, ^39^, ^40^ Figure 1 details the study screening and selection flow diagram.

### Study population and baseline characteristics

A total of 8 RCTs were included in the quantitative analysis, comprising 14,812 patients with CAD (ACS or chronic coronary syndrome).^22^, ^23^, ^24^, ^25^, ^26^, ^27^, ^28^, ^29^ Of these, 7,670 underwent IVI-guided PCI, while 7,142 underwent angiography-guided PCI. Follow-up durations ranged from 1 to 5 years, with a weighted mean follow-up of 1.9 years (Table 1). The majority of the study population was male (11,271 [76.1%]), with women representing a smaller proportion (3,541 [23.9%]). The mean age of participants was 64.6 years. Baseline comorbidities, including hypertension, diabetes, and dyslipidemia, were evenly distributed across groups (Table 2). All studies were multicenter and utilized DES for PCI.^22^, ^23^, ^24^, ^25^, ^26^, ^27^, ^28^, ^29^ Unstable angina (33.6%) and stable angina (33.1%) were the most common indications for PCI (Table 1). Definition of MACE varied across studies as detailed in Table 1.

**Table 1:** Characteristics of Included Studies

Study	Year	Comparison	Location	Number of Centers	Sample Size	Follow-Up (Months)	Stent Type	MACE Definition
IVUS-XPL	2020	ICA vs IVUS	East Asia	Multicenter	1,400 (700 vs 700)	60	Everolimus-eluting stent	Cardiac death, target lesion-related myocardial infarction, or ischemia-driven target lesion revascularization
ULTIMATE	2021	ICA vs IVUS	East Asia	Multicenter	1,448 (724 vs 724)	36	Second-generation DES	Cardiac death, target vessel myocardial infarction, and clinically driven target vessel revascularization
RENOVATE-COMPLEX-PCI	2023	ICA vs IVI	East Asia	Multicenter	1,639 (547 vs 1,092)	36	Polymer-coated everolimus-eluting stents	Cardiac death, target vessel-related myocardial infarction, or clinically driven target vessel revascularization
OCTOBER	2023	ICA vs OCT	Europe	Multicenter	1,201 (601 vs 600)	24	Xience everolimus-eluting stent	Cardiac death, target lesion myocardial infarction, or ischemia-driven target lesion revascularization
ILUMIEN IV	2024	ICA vs OCT	Europe, North America, Oceania, and East Asia	Multicenter	2,487 (1,254 vs 1,233)	24	Biocompatible polymer-coated everolimus-eluting stents (XIENCE, Abbott Vascular)	Cardiac death, target vessel myocardial infarction, or ischemia-driven target vessel revascularization
IVUS ACS	2024	ICA vs IVUS	Asia and Europe	Multicenter	3,505 (1752 vs 1753)	12	Second-generation DES	Cardiac death, target vessel myocardial infarction, or clinically driven target vessel revascularization
OCCUPI	2024	ICA vs OCT	South Korea	Multicenter	1,604 (801 vs 803)	12	Everolimus-eluting stents (XIENCE Alpine or XIENCE Sierra, Abbott Vascular, Chicago, IL, USA)	Cardiac death, myocardial infarction, stent thrombosis, or ischemia-driven target vessel revascularization
GUIDE DES	2024	QCA vs IVUS	Korea	Multicenter	1,528 (763 vs 765)	12	DES	Cardiac death, target vessel myocardial infarction, or ischemia-driven target lesion revascularization

DES = drug-eluting stent; GUIDE DES = Quantitative Coronary Angiography vs Intravascular Ultrasound Guidance for Drug-Eluting Stent Implantation, NCT02978456; ICA = invasive coronary angiography; ILUMIEN IV = Optical Coherence Tomography Guided Coronary Stent Implantation Compared to Angiography: A Multicenter Randomized Trial in PCI, NCT03507777; IVUS = intravascular ultrasound; IVUS ACS = 1-month vs 12-month DAPT for ACS Patients Who Underwent PCI Stratified by IVUS: IVUS-ACS and ULTIMATE-DAPT Trials, NCT03971500; IVUS-XPL = Impact of Intravascular Ultrasound Guidance on the Outcomes of Xience Prime Stents in Long Lesions [IVUS-XPL Study] Retrospective and Prospective Follow-Up Study, NCT03866486; OCT = optical coherence tomography; OCTOBER = European Trial on Optical Coherence Tomography Optimized Bifurcation Event Reduction, NCT03171311; OCCUPI = Optical Coherence Tomography-guided Coronary Intervention in Patients with Complex Lesions, NCT03625908; QCA = quantitative coronary angiography; RENOVATE-COMPLEX-PCI = Randomized Controlled Trial of Intravascular Imaging Guidance vs Angiography-Guidance on Clinical Outcomes after Complex Percutaneous Coronary Intervention, NCT03381872; ULTIMATE = Intravascular Ultrasound Guided Drug-Eluting Stents Implantation in “All-Comers” Coronary Lesions, NCT02215915.

**Table 2:** Baseline Characteristics of Included Studies

	Comparison	Age, y	Male, %	HTN, %	Dyslipidemia	DM	Current Smoker	LVEF, %	Prior MI	Prior PCI	Prior CABG	Stable Angina	UA	AMI
IVUS-XPL	IVUS vs Angio	63/63	69/69	65/63	67/65	32/38	22/23	62.8/62.3	5/5	11/10	3/3	49/52	36/32	15/17
ULTIMATE	IVUS vs Angio	65.2/65.9	73.9/73.2	70.7/72	53.7/55.2	30/31.2	NA	NA	NA	NA	NA	NA	NA	78.6/78.3
RENOVATE-COMPLEX-PCI	IVI vs Angio	65.3/66.0	79.6/78.8	62.5/59	51.3/51.2	39/45	19.4/17.4	58.4/59.3	6.9/7.7	24.5/23.2	N/A	48.7/50.3	33.1/31.6	36.7/35.9
OCTOBER	OCT vs Angio	66.4/66.2	78.8/79	70.3/74.5	76/78.4	17.2/16.1	12.8/14.1	59.5/58	28.3/30	40.7/42.8	1.2/1.5	55/53.4	8.8/9.7	36.2/37
ILUMIEN IV	OCT vs Angio	65.6/65.7	79.8/76.5	71.3/74.8	66.3/69.4	36.3/34.9	20/19.6	55.1/55	21.3/26	14.7/14.4	5.3/4.4	29/30.9	29.9/28.6	26.9/25.1
IVUS ACS	ICA vs IVUS	62/63	73.3/74.1	62.9/62.2	67.7/69.8	31.6/31.5	28.5/27.8	62/62	8.7/8.8	10.2/10.2	0.2/0.2	0/0	39.9/41.4	60.1/58.6
OCCUPI	OCT vs Angio	64/64	80/80	58/56	85/83	3/4	19/20	59.5/59.7	40/42	21/20	1/2	49/53	31/27	21/20
GUIDE DES	QCA vs IVUS	64.1/64.6	75.2/81.3	62.9/63.8	85.8/84.8	33.7/31.0	26.6/23.3	NA	6.2/7.3	15.3/15.6	0.9/0.8	71.3/70.3	NA	28.7/29.7

Values are mean or percent.

AMI = acute myocardial infarction; Angio = angiography; CABG = coronary artery bypass grafting; DM = diabetes mellitus; HTN = hypertension; IVI = intravascular imaging; LVEF = left ventricular ejection fraction; MI = myocardial infarction; NA= not available; PCI = percutaneous coronary intervention; UA = unstable angina; other abbreviations as in Table 1.

### Trends in inclusion of male and female patients in RCTS

**Figure 2:**
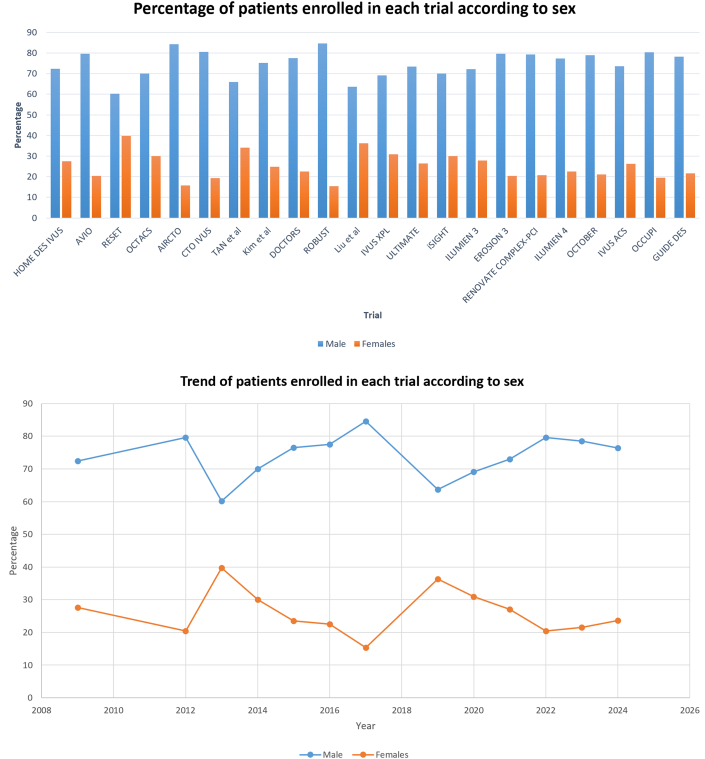
Trend of Patients Enrolled in Prior Trials According to Sex

The bar chart in Figure 2A illustrates the distribution of male and female participants across individual trials. The chart shows that male participants consistently accounted for 60 to 80% of the study population. In contrast, the proportion of female participants remained below 40% in all studies. A temporal trend analysis (Figure 2B) revealed that male representation in trials fluctuated between 70% and 80% over time, with minimal variation. There was consistently low female representation at <30% in most studies, with a handful enrolling up to 40% female patients.

### Outcomes

**Figure 3:**
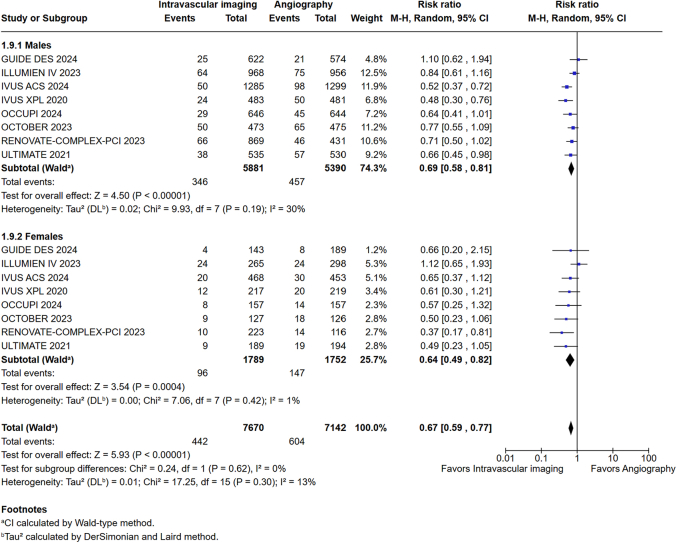
MACE in Males and Females Undergoing PCI for Coronary Lesions M-H = Mantel-Haenszel; MACE = major adverse cardiovascular events; PCI = percutaneous coronary intervention.

This meta-analysis demonstrated a comparable reduction in MACE in both men (RR: 0.69; 95% CI: 0.58-0.81; *P* < 0.001; I^2^ = 30%) (Figure 3) and women (RR: 0.64; 95% CI: 0.49-0.82; *P* < 0.001; I^2^ = 1%) (Figure 3) undergoing IVI-guided PCI compared to angiography alone for all coronary lesions (complex and noncomplex lesions). No significant interaction was observed between sex and MACE reduction with IVI guidance in these patients (*P*_interaction_ = 0.62) (Figure 3).

### Subgroup analyses

**Figure 4:**
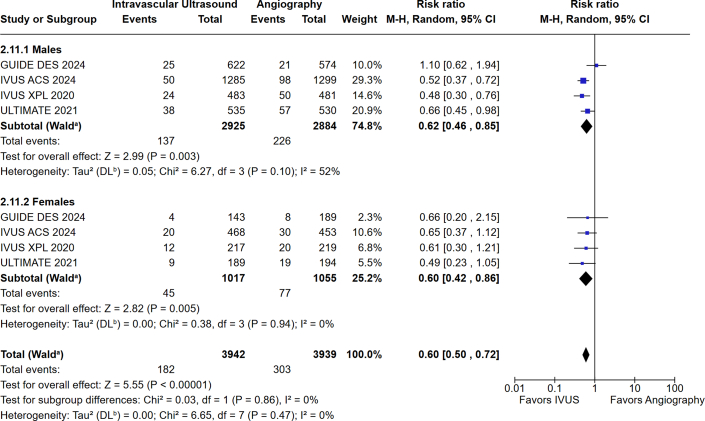
MACE in Males and Females Undergoing IVUS-Guided Vs Angiography-Guided PCI IVUS = intravascular ultrasound; other abbreviations as in Figure 3.

**Figure 5:**
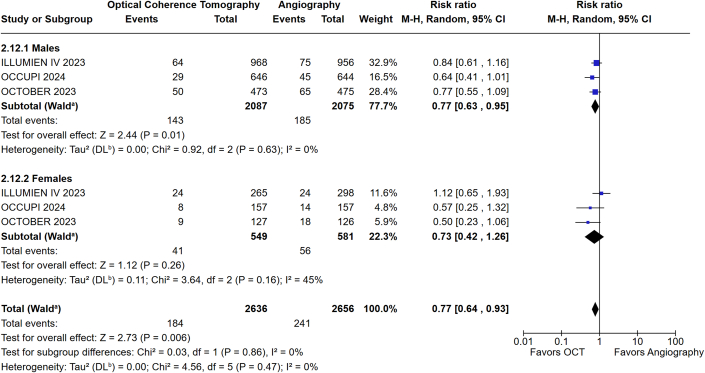
MACE in Males and Females Undergoing OCT-Guided Vs Angiography-Guided PCI OCT = optical coherence tomography; other abbreviations as in Figure 3.

Subgroup analysis based on IVI modality showed a reduction in MACE with IVUS-guided PCI among men (RR: 0.62; 95% CI: 0.46-0.85; *P* = 0.003; I^2^ = 52%) and women (RR: 0.60, 95% CI: 0.42-0.86; *P* = 0.005; I^2^ = 0%) compared with angiography alone. However, the *P* value of interaction was not significant (*P*_interaction_ = 0.86) (Figure 4). OCT guidance was associated with a significant reduction in MACE in men (RR: 0.77; 95% CI: 0.63-0.95; *P* = 0.01; I^2^ = 0%) but not women (RR: 0.73; 95% CI: 0.42-1.26; *P* = 0.26; I^2^ = 45%) compared to angiography alone. The *P* value of interaction was not significant (*P*_interaction_ = 0.86) (Figure 5).

**Figure 6:**
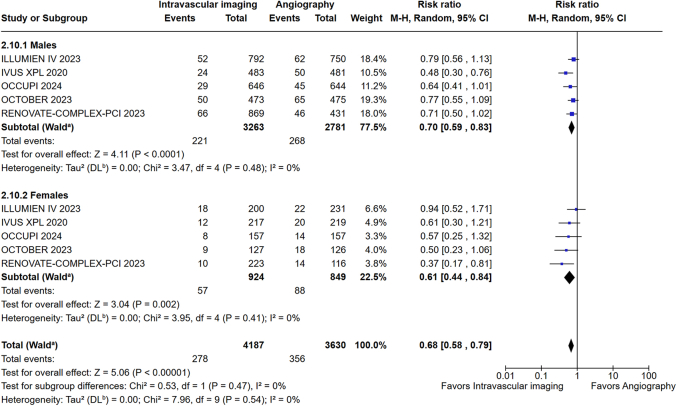
MACE in Males and Females Undergoing PCI for Complex Lesions Abbreviations as in Figure 3.

Among patients undergoing PCI for complex coronary lesions, IVI-guided PCI was associated with a reduction in MACE in both men (RR: 0.70; 95% CI: 0.59-0.83; *P* < 0.001; I^2^ = 0%) and women (RR: 0.61; 95% CI: 0.44-0.84; *P* = 0.002; I^2^ = 0%) compared to angiography alone (Figure 6). No significant interaction was observed between both sexes and IVI guidance among patients who underwent PCI for complex lesions (*P*_interaction_ = 0.47) (Figure 6). No heterogeneity was observed for all analyses performed. The overall risk of bias in the included trials was assessed using the Cochrane Collaboration tool and found to be low (Supplemental Figure 1). Funnel plot analysis revealed no evidence of publication bias (Supplemental Figure 2).

## Discussion

This systematic review and meta-analysis evaluated sex-specific clinical outcomes of IVI-guided vs angiography-only-guided PCI for coronary lesions (patients presenting with ACS and chronic coronary syndrome). The key findings include: 1) IVI-guided PCI significantly reduced MACE for both men and women compared with angiography alone with no sex interaction; 2) no significant interaction was observed between sex and MACE reduction with either IVUS or OCT guidance (although women failed to benefit from MACE reduction with OCT guidance); and 3) women remain persistently underrepresented in IVI-guided PCI trials (Central Illustration).

**Central Illustration:**
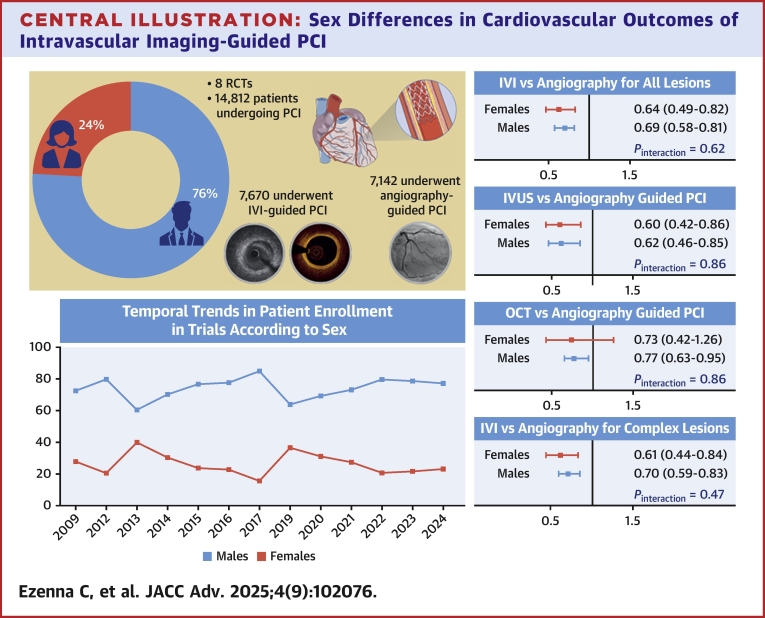
Sex Differences in Cardiovascular Outcomes of Intravascular Imaging-Guided PCI IVI = intravascular imaging; RCT = randomized controlled trials; other abbreviations as in Figure 3, Figure 4, Figure 5.

Our analysis reinforces the clinical efficacy of IVI-guided PCI in reducing MACE in both men and women compared with angiography-guided PCI. This is particularly relevant given the historical evidence of worse cardiovascular outcomes in women following PCI, often attributed to delayed diagnosis, atypical presentations of CAD, delay in revascularization, less ticagrelor use, later age of ACS presentation with more comorbidities, and disparities in health care access.^5^, ^6^, ^7^^,^^41^^,^^42^ Additionally, sex-based anatomical and pathophysiological differences, such as smaller coronary artery diameters, a higher prevalence of plaque erosion, and distinct vascular response to injury, have been shown to contribute to suboptimal PCI outcomes in women.^8^^,^^43^ The smaller-caliber coronary arteries of women may increase the risk of stent underexpansion and malapposition; both key predictors of restenosis and stent thrombosis.^2^ IVI facilitates accurate vessel sizing, less malapposition, and optimized stent deployment and expansion, helping to mitigate these risks.^2^ The high-resolution imaging of OCT further aids in identifying edge dissections and geographic miss that may go unnoticed with angiography alone.^2^^,^^26^^,^^38^ In our meta-analysis, these benefits translate to 31% and 36% relative reductions in MACE among men and women, respectively, further supporting the role of IVI in improving outcomes irrespective of sex. Our meta-analysis did not adjust for patient-level factors, and thus, the observed benefits of IVI guidance should not be interpreted as sufficient to fully address these systemic inequities. Rather, IVI should be viewed as one potential tool among many to optimize PCI outcomes in women, particularly in anatomically or procedurally challenging cases.

Second, we found no interaction between sex and MACE reduction with IVI guidance, indicating that both sexes derive substantial clinical benefits from IVI guidance. Prior studies report higher unadjusted rates of MACE and mortality in women after PCI, though such differences often attenuate after adjusting for baseline comorbidities.^8^^,^^44^^,^^45^ However, a recent review by Sambolia et al noted that women remained at higher risk for MACE post-PCI even after multivariable adjustment.^46^ Of note, a sex-stratified subgroup analysis of the RENOVATE COMPLEX-PCI (Randomized Controlled Trial of Intravascular Imaging Guidance vs Angiography-Guidance on Clinical Outcomes after Complex Percutaneous Coronary Intervention) trial demonstrated that IVI-guided PCI significantly reduced target vessel failure (TVF) in both sexes with a more pronounced effect in women (HR: 0.34; 95% CI: 0.15-0.78) compared to men (HR: 0.72; 95% CI: 0.49-1.05).^47^ Conversely, among patients treated with angiography guidance, TVF rates were higher in women than in men (14.5% vs 11.7%), whereas IVI guidance was associated with lower TVF rates in women compared to men, although these outcomes were not formally compared.^47^ The reduction was primarily driven by a 76% reduction in target vessel revascularization in women (*P* = 0.02) vs a 24% reduction in target vessel revascularization in men (*P* = 0.41) who underwent IVI-guided PCI compared to angiography alone.^47^ While the interaction by sex was not statistically significant, these results suggest that IVI may offer particular benefits in mitigating the longstanding sex-based differences in PCI outcomes.^47^

Our analysis found no significant interaction between sex and reduction in MACE with either IVUS or OCT guidance. The lack of statistical significance with OCT guidance in women may be attributed to the disproportionately smaller sample size (women = 549; men = 2,087) and the consequent reduced statistical power, as supported by the nonsignificant *P* value of interaction. This observation is in keeping with findings from the OCTIVUS (Optical Coherence Tomography Versus Intravascular Ultrasound Guided Percutaneous Coronary Intervention; NCT03394079) trial, which reported similar clinical benefits from both imaging modalities across sexes.^48^ In contrast, a small OCT-focused registry comprising 159 men and 47 women found that women had twice the rate of proximal coronary dissections during PCI compared to men (30.6% vs 15.6%; *P* = 0.02).^49^ However, the female sex was not independently associated with dissection risk after multivariable analysis.^49^ Chandrasekhar et al proposed that the observed difference may be related to the smaller vessel size commonly observed in women, which may influence the choice and feasibility of imaging modalities during PCI.^50^ Furthermore, the use of absolute minimal lumen area thresholds, such as the 3 mm^2^ cutoff commonly employed in IVUS-guided PCI in real-world practice, could result in higher PCI rates in women, whose smaller vessels are more likely to meet this threshold even in the setting of lower plaque burden.^51^

Despite the demonstrated benefits of IVI-guided PCI in women, our meta-analysis revealed a persistent underrepresentation of women in IVI trials, with only 23.9% of participants being female. This disparity raises concerns about the generalizability of trial findings, especially in the context of sex-specific differences in coronary anatomy and physiology, as highlighted above.^8^ Contemporary barriers likely contribute to this underrepresentation, including lower referral rates for invasive procedures, higher caregiving responsibilities, and logistical challenges that disproportionately affect women.^52^ Cultural and behavioral factors may further exacerbate this underrepresentation, as women may perceive greater risk associated with clinical trial participation and, consequently, may require additional reassurance and support.^53^, ^54^, ^55^ Supporting evidence from Scott et al and Ding et al reinforces our findings by showing that women are disproportionately excluded from cardiovascular trials, particularly those involving heart failure, CAD, and ACS.^56^^,^^57^ Reassuringly, a recent study by Ismayl et al showed comparable IVI use during PCI between men and women after adjusting for potential confounders.^13^ It is also important to note that the higher overall burden of CAD in men may partially contribute to the enrollment gap.^58^ Nevertheless, efforts to improve the representation of women in RCTs are critical to ensure that clinical guidelines reflect the unique needs of both sexes. Proposed strategies to promote equitable enrollment include sex-stratified recruitment quotas, mandated sex-based outcome analyses, and practical support measures such as transportation assistance, childcare provision, and culturally tailored educational resources.^59^^,^^60^ Ultimately, increasing female representation in RCTs may accelerate the adoption of IVI technologies in clinical practice, resulting in improved cardiovascular outcomes in this historically high-risk population.

### Strengths and limitations

Our meta-analysis provides some valuable insight into sex-specific outcomes of IVI-guided PCI in patients with both ACS and chronic coronary syndrome, which makes it the first comprehensive synthesis of RCTs on this topic. By including data from 8 high-quality RCTs, our study offers both comprehensive and robust evidence supporting the utilization of IVI-guided PCI for both men and women. The large sample size of 14,812 patients strengthens our findings while also allowing us to perform meaningful sex-stratified analyses. The use of random-effects models further strengthened our results by accounting for variability across individual studies, thus improving the overall generalizability of our findings.

Despite these strengths, our study has notable limitations. First, women comprised only a small percentage (23.9%) of the study population, which may limit the generalizability of the findings to a broader real-world female population. Second, differences in MACE definitions used by individual studies may have introduced some heterogeneity in the pooled analysis. Third, the exclusion of nonrandomized studies, particularly large-scale registries with higher female enrollment, may have resulted in the loss of valuable data that could further inform sex-specific outcomes. Fourth, some trials included in our analysis were conducted over a decade ago and utilized old-generation DES, which may limit the applicability of our findings to contemporary practice. Fifth, the enrolled populations in the included RCTs were often highly selected and may not fully capture the diversity and complexity of patients treated in routine clinical settings. Sixth, by excluding studies lacking sex-specific outcomes from our primary analysis, we may have omitted relevant data that could have potentially altered our findings. Finally, this meta-analysis was conducted at the study level rather than the patient level, which limited our ability to further explore study outcomes to better identify the specific drivers of MACE risk for men and women.

## Conclusions

Women remain underrepresented in IVI-guided PCI trials, despite a well-established pattern of suboptimal cardiovascular outcomes compared to men (partly due to anatomical differences, delayed presentation, structural barriers, etc.). This meta-analysis of RCTs demonstrates that IVI-guided PCI significantly reduces MACE in both men and women compared with angiography alone. There were no observed sex interactions in the magnitude of benefit with IVI guidance. These findings highlight the potential benefits of IVI as a practical, evidence-based tool capable of closing the long-standing sex gaps in coronary intervention. Addressing female underrepresentation in IVI-guided RCTs in future research will provide more definitive evidence on the sex-specific efficacy of IVI-guided PCI, which in turn may promote more equitable cardiovascular care for men and women.

Perspectives **COMPETENCY IN MEDICAL KNOWLEDGE:** In this meta-analysis, IVI-guided PCI significantly reduced MACE in both men and women compared with angiography alone, with no significant sex interaction in the magnitude of benefit. However, women remain underrepresented in IVI trials, comprising only 23.9% of the study population. These findings support the routine use of IVI to optimize PCI outcomes across sexes and highlight the need to address persistent sex disparities in clinical trial enrollment.

**TRANSLATIONAL OUTLOOK:** Future research should prioritize greater inclusion of women in IVI trials (particularly those evaluating OCT) and ensure sex-stratified analyses to guide evidence-based, equitable clinical practice. Continued efforts to standardize IVI use and assess its impact in anatomically complex or high-risk female patients are essential to narrowing the sex gap in coronary intervention outcomes.

## Funding support and author disclosures

Dr Goldsweig has consulted for Philips, Inari Medical, and Conformal Medical; and has done speaking for Philips and Edwards Lifesciences. Dr Nanna has received unrelated current research support from the 10.13039/100005485American College of Cardiology Foundation supported by the George F. and Ann Harris Bellows Foundation, the Patient-Centered Outcomes Research Institute (10.13039/100006093PCORI), the Yale 10.13039/100031147Claude D. Pepper Older Americans Independence Center (P30AG021342), and the 10.13039/100000049National Institute on Aging (K76AG088428)—personal fees from Heartflow, Inc, Merck, and Novo Nordisk. All other authors have reported that they have no relationships relevant to the contents of this paper to disclose.
